# Water-Soluble Fullerenol C_60_(OH)_36_ toward Effective Anti-Air Pollution Induced by Urban Particulate Matter in HaCaT Cell

**DOI:** 10.3390/ijms20174259

**Published:** 2019-08-30

**Authors:** Chiang-Wen Lee, Miao-Ching Chi, Kuo-Ti Peng, Yao-Chang Chiang, Lee-Fen Hsu, Yi-Ling Yan, Hsing-Yen Li, Ming-Chun Chen, I-Ta Lee, Chian-Hui Lai

**Affiliations:** 1 Department of Nursing, Division of Basic Medical Sciences, and Chronic Diseases and Health Promotion Research Center, Chang Gung University of Science and Technology, Puzi City, Chiayi County 613, Taiwan; 2 Research Center for Industry of Human Ecology and Research Center for Chinese Herbal Medicine, Chang Gung University of Science and Technology, Guishan District, Taoyuan City 333, Taiwan; 3 Department of Orthopaedic Surgery, Chang Gung Memorial Hospital, Puzi City, Chiayi County 613, Taiwan; 4 Department of Respiratory Care, Chang Gung University of Science and Technology, Puzi City, Chiayi County 613, Taiwan; 5 Division of Pulmonary and Critical Care Medicine, Chiayi Chang Gung Memorial Hospital, Kaohsiung 833, Taiwan; 6 College of Medicine, Chang Gung University, Guishan District, Taoyuan City 333, Taiwan; 7 Division of Neurosurgery, Department of Surgery, Chang Gung Memorial Hospital, Puzi City, Chiayi County 613, Taiwan; 8 Graduate Institute of Biomedical Engineering, National Chung Hsing University, Taichung 402, Taiwan; 9 Department of Medicinal and Applied Chemistry, Kaohsiung Medical University, Kaohsiung 807, Taiwan; 10 School of Dentistry, College of Oral Medicine, Taipei Medical University, Taipei 110, Taiwan

**Keywords:** fullerenol, particulate matter, reactive oxygen species, human keratinocyte cell, antioxidant, anti-inflammation

## Abstract

Particulate matter (PM), a widespread air pollutant, consists of a complex mixture of solid and liquid particles suspended in air. Many diseases have been linked to PM exposure, which induces an imbalance in reactive oxygen species (ROS) generated in cells, and might result in skin diseases (such as aging and atopic dermatitis). New techniques involving nanomedicine and nano-delivery systems are being rapidly developed in the medicinal field. Fullerene, a kind of nanomaterial, acts as a super radical scavenger. Lower water solubility levels limit the bio-applications of fullerene. Hence, to improve the water solubility of fullerene, while retaining its radical scavenger functions, a fullerene derivative, fullerenol C_60_(OH)_36_, was synthesized, to examine its biofunctions in PM-exposed human keratinocyte (HaCaT) cells. The PM-induced increase in ROS levels and expression of phosphorylated mitogen-activated protein kinase and Akt could be inhibited via fullerenol pre-treatment. Furthermore, the expression of inflammation-related proteins, cyclooxygenase-2, heme oxygenase-1, and prostaglandin E2 was also suppressed. Fullerenol could preserve the impaired state of skin barrier proteins (filaggrin, involucrin, repetin, and loricrin), which was attributable to PM exposure. These results suggest that fullerenol could act against PM-induced cytotoxicity via ROS scavenging and anti-inflammatory mechanisms, and the maintenance of expression of barrier proteins, and is a potential candidate compound for the treatment of skin diseases.

## 1. Introduction

Exposure to air particulate matter (PMs) through inhalation is correlated with pulmonary dysfunction, cardiovascular disease, atherosclerosis, and hepatic fibrogenesis, and with a higher level of morbidity and mortality [1,2]. Cumulative data from epidemiological studies have shown that exposure to air pollutants could lead to an increase in cardiovascular ischemic events (out-of-hospital cardiac arrests and ischemic heart disease) and enhance atherosclerosis; their possible pathogeneses were also investigated [2,3,4,5]. PM exposure affected the functioning of pulmonary and cardiovascular systems, and is also associated with skin aging, skin cancer, and inflammatory responses to allergic skin conditions (atopic dermatitis, eczema, psoriasis, or acne) [6]. The possible pathological mechanisms for PM-induced skin diseases are linked to an increase in reactive oxygen species (ROS) level and inflammatory activity, and a loss of barrier proteins [7]. PMs are complex, heterogeneous mixtures of polyaromatic hydrocarbons, metals, organic toxins, and biological materials. In general, PMs can be grouped into four classes based on their size, as ultrafine particles (PM < 0.1 μm), fine particles (PM < 2.5 μm), coarse particles (PM 2.5–10 μm), and thoracic particles (PM >10 μm) [4]. Among these air pollutant particles, the fine particles (PM_2.5_) have the longest atmospheric half-life of a few days to weeks [4]. PMs can induce the generation of ROS, which potentially triggers oxidative stress and subcellular organelle dysfunction in the cell, and triggers apoptosis by causing DNA damage or lipid peroxidation [8,9]. PM_2.5_ induced endoplasmic reticulum stress, mitochondrial swelling, and autophagy, and caused apoptosis in the human keratinocyte (HaCaT) model and mouse skin tissue [8]. Previously, we had also studied PM-induced toxic effects that were attributable to inflammatory and oxidative stress mechanisms in vitro and in vivo [10].

Fullerene, a nanomaterial, has valuable applications in the biomedicine field. It has been used in skin whitening, sunscreen, or antiaging products in the dermatologic and cosmetic fields, because of its extremely high reactivity to radical species [11]. Since 1995, when the FDA first approved a nano-drug, nanomedicine has constantly revolutionized the treatment of several diseases [12,13,14]. Fullerene, first discovered in 1985, is a kind of carbon allotrope, and could form a hollow sphere, such as that observed in a soccer ball [15]. Fullerene can be best described as a polygonal structure with 60 carbon atoms that have 60 vertices and 32 faces, which are responsible for its stable configuration [16]. Fullerene is a super radical scavenger with a single C_60_ molecule that could reportedly include up to 34 methyl radicals onto itself [17]. Fullerene has biomedical functions, such as the induction of photo-induced cytotoxicity for DNA cleavage [18], and antioxidant, anticancer, and antimicrobial activities [19]. However, the poor water solubility of fullerene limits its bio-application; therefore, the water-soluble polyhydroxylated fullerene derivative, fullerenol C_60_(OH)_36_, was introduced in the current study, to perform a bioassay. Fullerenol can reduce the toxic effects of certain hazardous toxicants [20], infarct volume, and cerebral inflammation during ischemic stroke in normotensive and hypertensive rats [21]. Besides, fullerenol C_60_(OH)_36_ can protect the human erythrocyte membrane against high-energy electrons [22]. The protective functions of fullerenol against PM-induced disease have not been studied yet.

We had previously developed both eupafolin [23] and eupafolin nanoparticle (NP) delivery systems [24] and a water-soluble trihydroxyisoflavone NP [25] system, to study their individual anti-oxidant and anti-inflammatory activities that prevented PM-induced skin inflammation. Our previous findings revealed that PM could mediate the AhR/p47 phox/NADPH oxidase pathway, and subsequently results in ERK1/2, p38/NF-κB and JNK/AP-1 activation. Finally, PM induces COX-2 protein expression and filaggrin downregulation. The upregulated expression of COX-2 and production of PGE2 might impair their ability to function as skin barriers [10]. PM could increase the level of oxidative stress to the cell and cause either serious irregularities in signal transduction or protein expression in cells; hence, because fullerene nanoparticles can act as an exceptional antioxidant; they have been applied in the dermatologic and cosmetic fields [11]. To continue our efforts to study the inhibition of PM-induced inflammation, we have tried to develop a water-soluble fullerene derivative, fullerenol C_60_(OH)_36_, as a new nanomedicine, and evaluated its anti-air pollution activity on HaCaT cells. We hypothesize that fullerenol C_60_(OH)_36_ might be used in cosmeceutical products or medicines that inhibit and prevent PM-induced skin injuries in the future. The levels of ROS, expression of phosphorylated mitogen-activated protein kinase, Akt, and inflammation-related proteins (cyclooxygenase-2, heme oxygenase-1, and prostaglandin E2) that were induced by PM were analyzed. Four specific skin barrier proteins (filaggrin, involucrin, repetin, and loricrin) were also studied.

## 2. Results

### 2.1. Synthesis and Characterization of Fullerenol C_60_(OH)_36_

**Figure 1:**
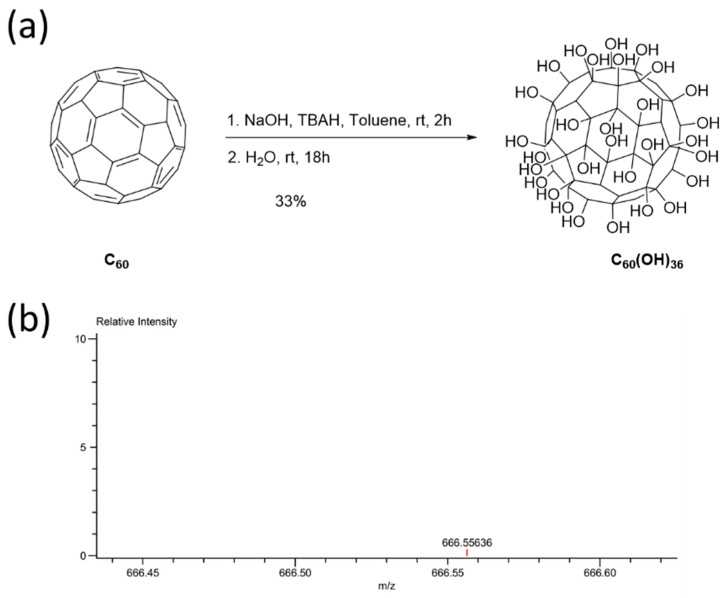
(**a**) Synthesis procedure for fullerenol C_60_(OH)_36_; (**b**) ESI-TOF MS spectral data.

Several methods can be used to synthesize fullerenols with different degrees of hydroxyl groups [26]. In this report, an alkaline reaction method was applied to synthesize fullerenol, C_60_(OH)_36_ [21]. The fullerene C_60_ was first dissolved in toluene, after which aqueous NaOH and TBAH solutions were added to the reaction mixture (Figure 1a). After the removal of toluene, water was added and the reaction was allowed to occur for 18 h. The aqueous phase was then evaporated. The crude sample was formed as a brown sludge that became precipitated upon the addition of methanol. The precipitate was finally washed with methanol several times, to remove traces of TBAH and NaOH, to obtain C_60_(OH)_36_. The molecular weight of fullerenol C_60_(OH)_36_ was confirmed by ESI-TOF MS.

**Figure 2:**
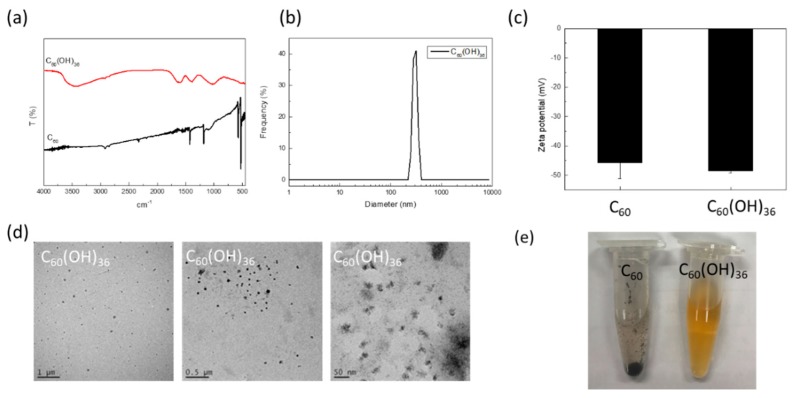
(**a**) FTIR, (**b**) DLS, (**c**) zeta-potential, and (**d**) TEM data; (**e**) images for fullerenol C_60_(OH)_36_ or fullerene C_60_, respectively.

Fullerenol C_60_(OH)_36_ was then characterized by fourier-transform infrared spectroscopy (FTIR), DLS, and TEM (Figure 2). The FTIR spectrum of fullerenol C_60_(OH)_36_ and the starting material, i.e., fullerene C_60_, are shown in Figure 2a. The FTIR spectrum of C_60_(OH)_36_ (Figure 2a) indicated a broad single at around 3400 cm^−1^, at which typical O–H stretching occurred for C_60_(OH)_36_. The starting material, fullerene C_60_, shows typical IR signals at ~1482, ~1180, ~575, and ~526 cm^−1^ [27]. The specific bands at 1620, 1370, and 1080 cm^−1^ are characteristic of C = C, C-O-H, and C-O absorption, respectively, on C_60_(OH)_36_. The hydrodynamic diameter of fullerenol C_60_(OH)_36_ is 283.5 ± 28.0 nm (Figure 2b). The zeta potentials of fullerene C_60_ and fullerenol C_60_(OH)_36_ in water are −45.7 ± 5.4 and −48.6 ± 0.6, respectively (Figure 2c). The TEM images showed the fullerenol C_60_(OH)_36_ particle morphology; its size was ~20–30 nm (Figure 2d). Besides, as shown in Figure 2e, fullerene C_60_ is insoluble in water and forms a precipitate in the Eppendorf tube; in contrast, fullerenol C_60_(OH)_36_ was completely suspended in the aqueous solution.

### 2.2. Fullerenol C_60_(OH)_36_ Blocked PM-Induced Cell Apoptosis

**Figure 3:**
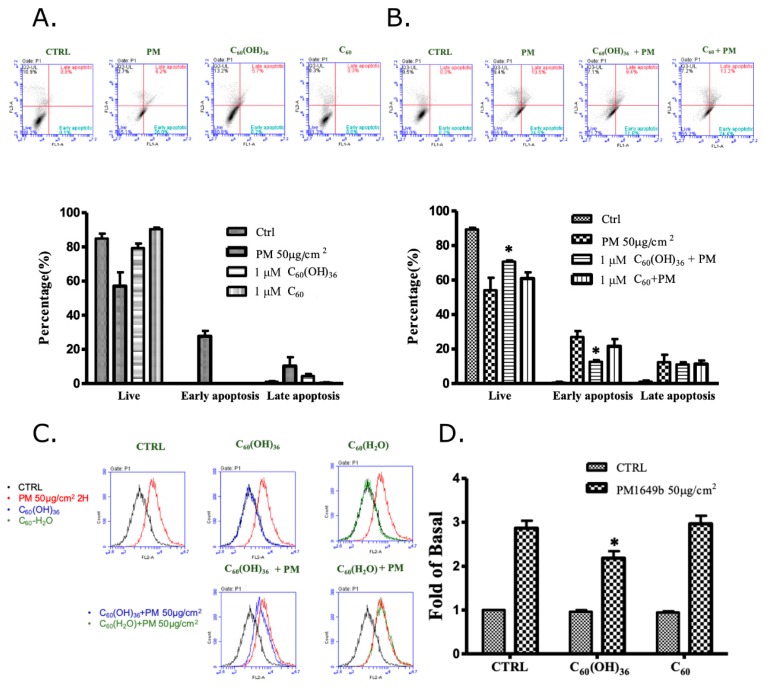
Effects of fullerenol (C_60_(OH)_36_), fullerene (C_60_), and PM on cell apoptosis in human skin keratinocyte (HaCaT) cells. Cells were treated with (**A**) fullerenol (C_60_(OH)_36_), fullerene (C_60_), or PM for 24 h, and pre-treated with (**B**) fullerenol (C_60_(OH)_36_) and fullerene (C_60_) (1 μM) for 1 h; then, cells were treated with PM (50 μg/cm^2^) for another 24 h, and values were measured via flow cytometry by Annexin V-fluorescein isothiocyanate (FITC)/propidium iodide staining. (**C**) The raw data and (**D**) the calculated values of caspase-3 activity, which were measured by flow cytometry, were presented. The upper panel of (**A**,**B**) shows the raw flow cytometry data, and the lower right quadrant of each raw figure indicates the percentage of cells undergoing early apoptosis. The bar graphs illustrate the cumulative counts observed using the flow cytometer. All data are expressed as means ± SEM values from at least three individual experiments. * *p* < 0.05, as compared to the values for the PM exposure group.

Annexin V-fluorescein isothiocyanate (FITC)/propidium iodide staining was used to detect apoptosis at an early stage in HaCaT cells, using flow cytometry. Annexin V is a Ca^2+^-dependent phospholipid-binding protein, which exhibits a high affinity towards phosphatidylserine, when exposed to the external cellular environment, at an early stage of apoptosis. Hence, annexin V could identify early stages of apoptosis more effectively than other assays that are based on nuclear changes (DNA fragmentation). Annexin V staining precedes the loss of membrane integrity, which also occurred after the later stages of cell death (apoptotic or necrotic processes). Therefore, propidium iodide is usually used in conjunction with Annexin V to identify intact cellular membranes. Hence, a combination of Annexin V and propidium iodide positive/negative signals could be used to identify early apoptosis, or the late stages of apoptosis and necrosis. In addition, Annexin V is commonly used for detecting apoptosis in the HaCaT cell [28,29]. As shown in Figure 3A, fullerenol (C_60_(OH)_36_) and water non-soluble fullerene (C_60_) did not affect the early apoptosis of HaCaT cells, while PM could accelerate early apoptosis in HaCaT cells. Pre-treatment with 1 μM of fullerenol (C_60_(OH)_36_) for 1 h could partially but significantly suppress PM (SRM 1649b, 50 μg/cm^2^)-triggered cell apoptosis, especially during early apoptosis, but could not do so when fullerene C_60_ was used (Figure 3B). Furthermore, the similar effects of fullerenol (C_60_(OH)_36_) were obtained in the caspase-3 activities assay (Figure 3C,D). These results indicate that pre-treatment with fullerenol (C_60_(OH)_36_) could partially suppress PM-induced apoptosis.

### 2.3. Fullerenol C_60_(OH)_36_ Suppressed PM-Triggered the Cellular and Mitochondrial Production of Reactive Oxygen Species 

**Figure 4:**
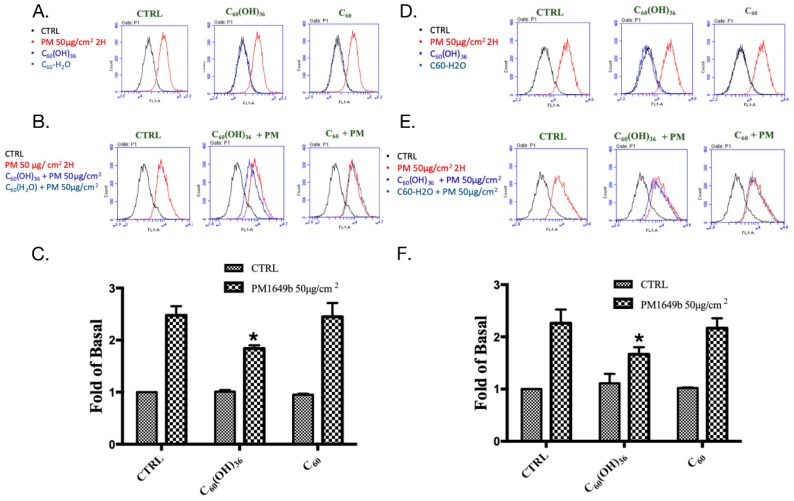
Cellular reactive oxygen species (ROS) production after treating HaCaT cells with fullerenol C_60_(OH)_36_, fullerene C_60_, and PM. (**A**) Raw flow cytometry data obtained after H2DCFDA staining of cells treated with fullerenol C_60_(OH)_36_, fullerene C_60_, or PM alone; (**B**) cells were treated with fullerenol C_60_(OH)_36_ or fullerene C_60_ for 1 h and then administered PM (SRM 1649b, 50 μg/cm^2^) for another 2 h. Signals were detected via H2DCFDA staining. (**C**) The bar graphs illustrate the cumulative counts of H2DCFDA flow cytometry results. (**D**) Raw flow cytometry data obtained after CellROX staining of cells treated with fullerenol C_60_(OH)_36_, fullerene C_60_, or PM alone; (**E**) cells were treated with fullerenol C_60_(OH)_36_ or fullerene C_60_ for 1 h and then administered PM for another 2 h. Signals were detected with CellROX staining. (**F**) The bar graphs illustrate the cumulative counts of CellROX flow cytometry results. All bar graph data were collected from at least three individual experiments and expressed as mean ± SEM values. * *p* < 0.05, as compared to the values for the PM exposure group.

**Figure 5:**
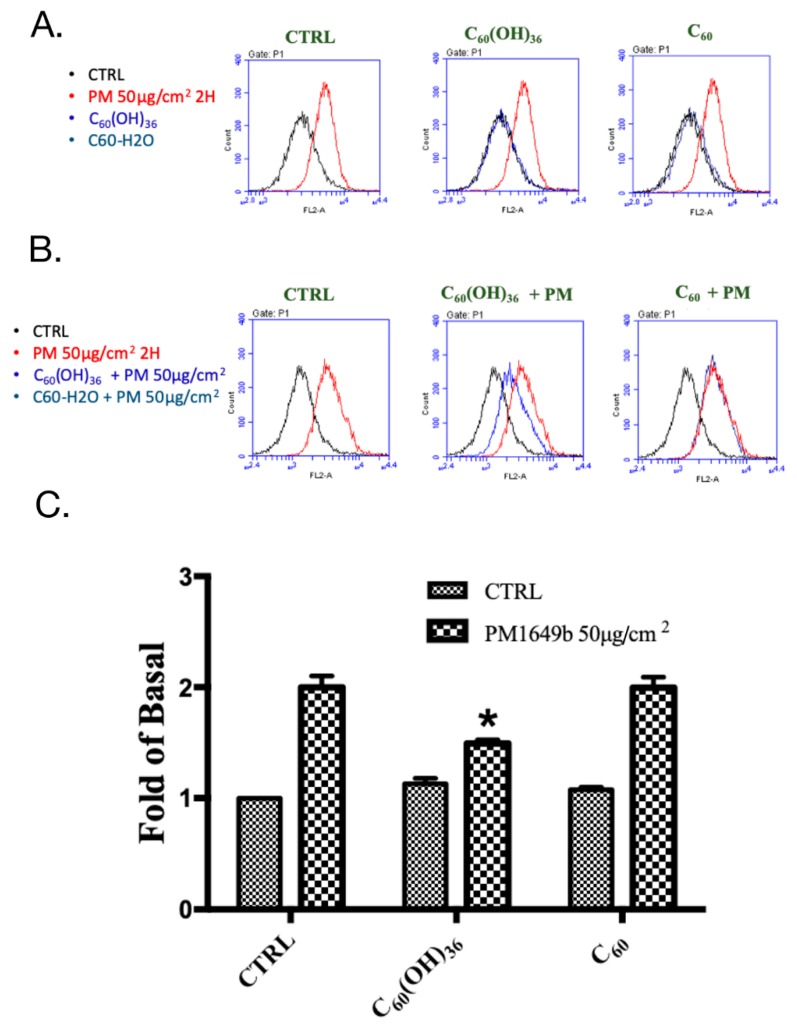
Effect of fullerenol C_60_(OH)_36_, fullerene C_60_, and PM on mitochondrial reactive oxygen species (ROS) production (determined by flow cytometry with MitoSOX staining) in HaCaT cells. (**A**) Raw flow cytometry data obtained along with MitoSOX staining data in cells treated with fullerenol C_60_(OH)_36_, fullerene C_60_, or PM alone. (**B**) Raw flow cytometry data obtained along with MitoSOX staining data in cells that received fullerenol C_60_(OH)_36_, or fullerene C_60_ (1 μM) for 1 h, after which PM (SRM 1649b, 50 μg/cm^2^) was administered for another 2 h. (**C**) The bar graph illustrates the cumulative counts of MitoSOX flow cytometry results. All bar graph data were collected from at least three individual experiments and expressed as mean ± SEM values. * *p* < 0.05, as compared to the values for the PM exposure group.

It is known that PM could increase ROS levels and subsequently cause diseases [5,10]. Moreover, a previous study has suggested that the presence of water-soluble fullerenes could enhance their ROS scavenging activity [30]. Hence, the effects of nanolization of fullerenol C_60_(OH)_36_ on ROSs production were investigated. In the study, three ROS probes were used in a flow cytometry system, to detect the different types and locations of ROS within cells. H2DCFDA was majorly used to measure the activity of hydroxyl and peroxyl groups. The CellROX Red dye does not bind to DNA; hence, it was localized in the cytoplasm. CellROX is weakly fluorescent, though it shows photostable fluorescence after ROS oxidation. Figure 4C shows that the presence of fullerenol C_60_(OH)_36_ and fullerene C_60_ in water did not change the ROS levels, as compared to those of the control group, upon measuring a cellular ROS determinant reagent, H2DCFDA. However, PM triggered a significant increase in the ROS level. Most importantly, fullerenol C_60_(OH)_36_ could significantly inhibit PM-caused ROS production, though fullerene C_60_ in water could not do so. Similar results were obtained after cell staining with another cellular ROS staining reagent, CellROX (Figure 4F). However, our results showed that fullerenol C_60_(OH)_36_ only partially suppressed the PM-induced increase in cellular ROS. Based on the detection ranges of assay kits, this might imply that other pathways, such as NO or CO pathways, influence ROS generation. ROS could be generated in cells as well as mitochondria. Hence, the mitochondrial ROS levels were then detected via flow cytometry and MitoSOX staining with similar fullerenol or fullerenes, and a PM-treated schedule for the cellular ROS assy. MitoSox is a red dye that is specifically sensitive to superoxides produced by mitochondria in live cells. As shown in Figure 5C, the ROS levels were increased by PM treatment, while the presence of fullerenol C_60_(OH)_36_ or fullerene C_60_ in water did not affect ROS production in mitochondria, as compared to that for the control group. Moreover, fullerenol C_60_(OH)_36_ (1 μM) pre-treatment could suppress PM-triggered mitochondrial ROS generation in HaCaT cells. The results indicate that the PM-induced production of cellular and mitochondria ROS (H_2_O_2_ and superoxide) could be prevented by pre-treatment with fullerenol C_60_(OH)_36_, but not with fullerene C_60_ in water.

### 2.4. Fullerenol C_60_(OH)_36_ Reduced the PM-Induced Phosphorylation of Mitogen-Activated Protein Kinases (MAPKs) and AKT Proteins in HaCaT Cells

**Figure 6:**
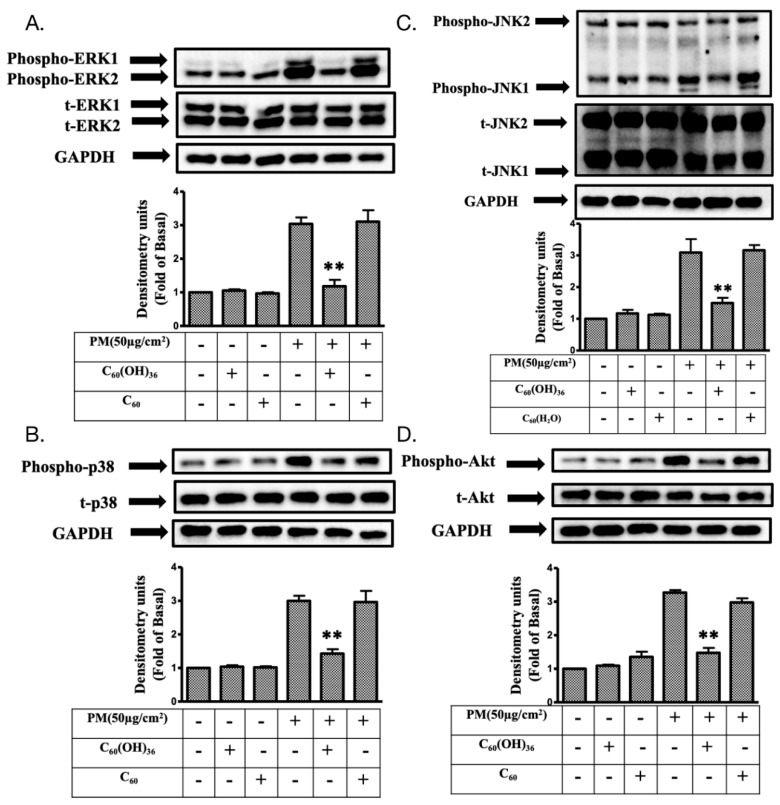
The changes in phosphorylated MAPK and Akt protein levels in fullerenol C_60_(OH)_36_ and PM-treated HaCaT cells. The expression levels of different kinases, including (**A**) extracellular signal-regulated kinase (ERK, p42/p44), (**B**) P38, (**C**) c-Jun N-terminal kinase (JNK), and (**D**) Akt were measured by immunoblotting in the HaCaT cells with or without providing pre-treatment with 1 μM of fullerenol C_60_(OH)_36_ or fullerene C_60_ for 1 h and with PM (SRM 1649b, 50 μg/cm^2^) for another 6 h. Glyceraldehyde 3-phosphate dehydrogenase (GAPDH) was used as a loading control. Blots were representative of three independent experiments, and the data were expressed as mean ± SEM values. ** *p* < 0.01, as compared to the values for the PM exposure group.

It has been demonstrated that mitogen-activated protein kinase (MAPK) and Akt (also called protein kinase B) levels could be regulated by ROS and could subsequently regulate various important cellular processes, such as transcription factor expression, cell proliferation, growth, and death [10,31]. Immunoblotting is a useful assay tool for the measurement of changes in protein expression levels. Hence, immunoblotting was used to determine the effects of fullerenol C_60_(OH)_36_ on PM-induced changes in expression levels of MAPK and Akt pathway proteins in HaCaT cells. As shown in Figure 6, the presence of fullerenol C_60_(OH)_36_ and fullerene C_60_ in water did not change the levels of total and phosphorylated proteins of extracellular signal-regulated kinases 1 and 2 (ERK, pp42 and pp44), P38, c-Jun N-terminal kinase (JNK), and Akt. However, higher expression levels of phospho-ERK, P38, JNK, and Akt were observed after the treatment of cells with PM for another 6 h. Furthermore, the PM-induced expression of phosphorylated MAPK and Akt proteins was significantly suppressed by pre-treatment with 1 μM of fullerenol C_60_(OH)_36_; these effects were not observed with fullerene C_60_ dissolved in water.

### 2.5. Fullerenol C_60_(OH)_36_ Suppressed PM-Induced Inflammatory Protein Expression

**Figure 7:**
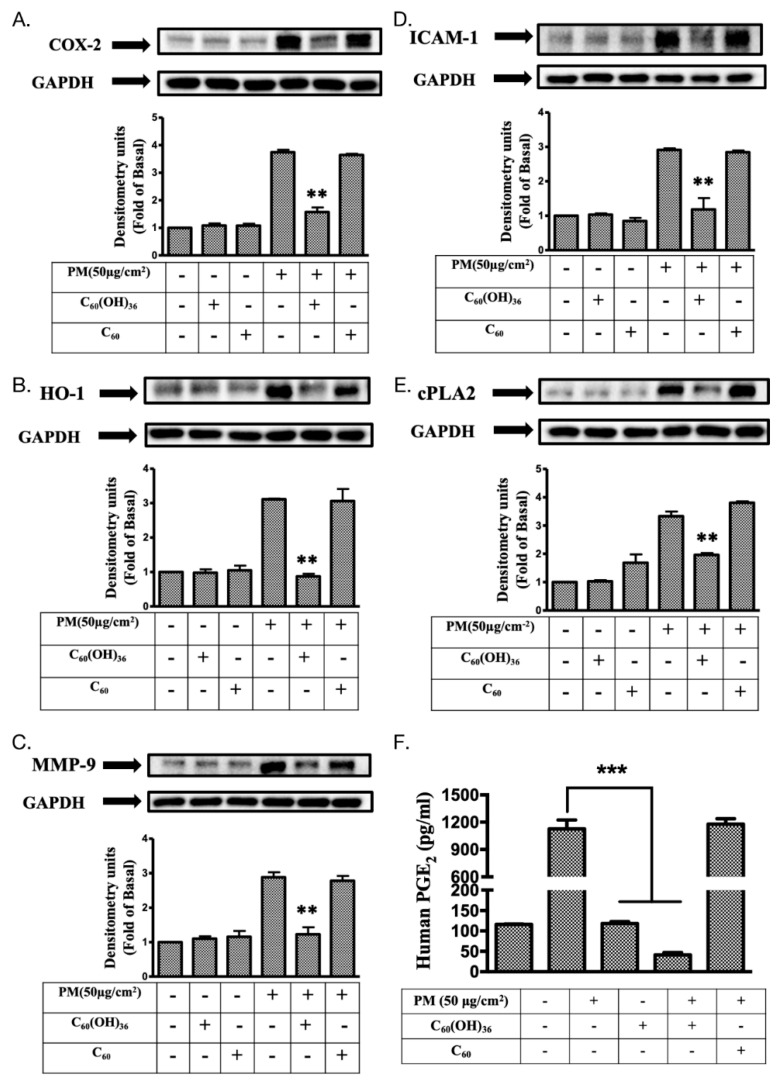
The changes in inflammation-related proteins in the fullerenol C_60_(OH)_36_ and PM-treated HaCaT cells. The effects of inflammation-related proteins, including (**A**) cyclooxygenase-2 (COX-2), (**B**) intercellular adhesion molecular-1 (ICAM-1), (**C**) heme oxygenase-1 (HO-1), (**D**) cytosolic phospholipase A2 (cPLA2), and (**E**) metalloproteinase-9 (MMP-9) were measured by immunoblotting, and (**F**) prostaglandin E2 (PGE2) levels were detected using an ELISA kit in HaCaT cells that did or did not receive pre-treatment with 1 μM fullerenol C_60_(OH)_36_ or fullerene C_60_ dissolved in water for 1 h, followed by PM (SRM 1649b, 50 μg/cm^2^) treatment for another 24 h. Glyceraldehyde 3-phosphate dehydrogenase (GAPDH) was used as a loading control during the immunoblotting process. Data were collected from at least three individual experiments and expressed as mean ± SEM values. * *p* < 0.01, as compared to the values for the PM exposure group.

Several inflammatory proteins could be induced by ROS, to cause cell or tissue injury, because of the effects of activation of cellular kinase pathways [10,32]. Here, various inflammatory proteins were selected for analysis via immunoblotting. The levels of cyclooxygenase-2 (COX-2), prostaglandin E2 (PGE2), intercellular adhesion molecular-1 (ICAM-1), heme oxygenase-1 (HO-1), cytosolic phospholipase A2 (cPLA2), and metalloproteinase-9 (MMP-9) were detected after PM or fullerene treatment. As shown in Figure 7, the base levels of COX-2, ICAM-1, HO-1, cPLA2, MMP-9, and PGE2 were not changed after treatment with fullerenol C_60_(OH)_36_ (1 μM) or fullerene C_60_ in water, as compared to the levels for the control group. Moreover, fullerenol C_60_(OH)_36_ could significantly block PM-induced COX-2, ICAM-1, HO-1, cPLA2, and MMP-9 protein expression and PGE2 production, but these phenomena were not observed in the group treated with fullerene C_60_ in water.

### 2.6. Fullerenol C_60_(OH)_36_ Could Maintain the Levels of Proteins Exhibiting Protective Effects towards PM-Exposed Keratinocytes

**Figure 8:**
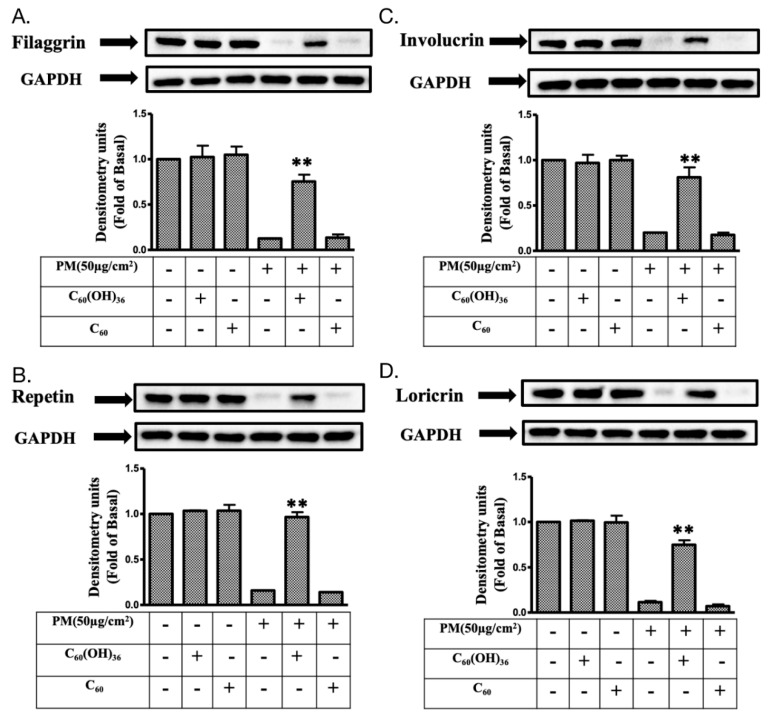
The expression levels of proteins exhibiting protective effects towards keratinocytes in fullerenol C_60_(OH)_36_ and PM-treated HaCaT cells. The effects of proteins with protective effects, including (**A**) filaggrin, (**B**) repetin, (**C**) involucrin, and (**D**) loricrin, were measured by immunoblotting in HaCaT cells that did or did not receive pre-treatment with fullerenol C_60_(OH)_36_ or fullerene C_60_ dissolved in water for 1 h and with PM (SRM 1649b, 50 μg/cm^2^) for another 24 h. Glyceraldehyde 3-phosphate dehydrogenase (GAPDH) was used as a loading control. Blots were representative of three independent experiments, and data are expressed as mean ± SEM values. * *p* < 0.01, as compared to the values for the PM exposure group.

Filaggrin, involuvrin, repetin, and loricrin are known to act as barriers against damage caused by PM exposure in keratinocytes [10,33]. In the above experiments, we have suggested that fullerenol C_60_(OH)_36_ could reduce the expression of proteins involved in inflammatory pathways via scavenging ROS production. We further investigated whether fullerenol C_60_(OH)_36_ protected PM-exposed HaCaT cells from protein loss. The immunoblotting method was applied in this experiment. As shown in Figure 8, significant suppressive effects were observed on the PM-induced loss of filaggrin, involuvrin, loricrin and repetin (at 24 h) after pre-treatment with 1 μM fullerenol C_60_(OH)_36_, but these effects were not observed with water-dissolved fullerenes C_60_. This indicates that fullerenol C_60_(OH)_36_ could maintain the levels of proteins exhibiting protective effects towards PM-exposed keratinocytes and act against PM-induced skin aging.

## 3. Discussion

Fullerene has been called a “radical sponge” because of its extremely high reactivity to radical species [11]. Because fullerene nanoparticles exhibit an exceptional antioxidant capacity, they have been applied in dermatologic and cosmetic fields [11]. In general, fullerene can be easily obtained in skin whitening, sunscreen, or antiaging products. However, the low water solubility of non-modified fullerene C_60_ limits its further use in bio-applications. Therefore, this study focuses on the synthesis of water-soluble fullerenes C_60_(OH)_36_ and investigates the levels of inflammation and oxidative stress induced by them in response to PM exposure. Water-soluble fullerenols C_60_(OH)_36_ were synthesized according to a previously described procedure that was modified slightly (Figure 1) [27,34]. FTIR measurement (Figure 2a) showed that fullerenol had been synthesized successfully, and that it had functional hydroxyl groups around 3400 cm^−1^ that clearly differed from the fullerene signal. The DLS data (Figure 2b) provides the hydrodynamic diameter ranges of fullerenol C_60_(OH)_36_ in water. TEM images can complementarily estimate the sizes and further enable the particle morphology of fullerenols C_60_(OH)_36_ to be visualized (Figure 2d).

Fullerenol can also show several antioxidant effects [27]. The antioxidant properties of fullerenol were first described for an anti-ROS-induced injury of the hippocampus in vitro [35], in animal models of intestinal ischemia-reperfusion [36], and even in cases of transplantation [37]. Fullerenol C_60_(OH)_36_ can protect the human erythrocyte membrane against high-energy electrons [22].

The inhalation of ambient PM generated because of industrialization and urbanization has adverse effects on human health [38,39] and causes problems such as pulmonary dysfunction, cardiovascular disease, atherosclerosis, and hepatic fibrogenesis, which increases the morbidity and mortality [1,2]. PM affects the visceral dysfunctions; in addition, previous studies have suggested that an increase in the PM concentration was highly associated with the occurrence and progression of various skin diseases attributable to oxidative stress and the activation of inflammatory pathways [40]. Studies showed that PM can cause abnormal ROS production and accumulation [5,10]. ROS affects the physiological and pathological functions of cells, tissues, and even the entire organism [41,42,43]. ROS could facilitate the occurrence of several cellular activities, such as cell-signaling transduction, homeostasis regulation, and phagocytosis, after which ROS could be eliminated by the scavenging system (enzymes, superoxide dismutase, catalase, lactoperoxidase, glutathione peroxidase, and peroxiredoxin) of cells under physiological conditions [44,45]. Under pathological conditions, high levels of ROS that were difficult to eliminate were generated; they caused oxidative stress in cells. Subsequently, cellular biomacromolecules (lipids, sugars, proteins, and DNA) were oxidized by ROS; those of the secondary product generated by ROS might cause more harmful damage than that observed in the initially formed ROS. Hence, the aggravation and exacerbation of several diseases and phenomena, such as inflammation, neurodegeneration, aging, and cancer, were observed [45,46,47,48,49].

Small molecules, such as vitamin C, α-tocopherol (vitamin E), and glutathione are well known as antioxidants that reportedly prevent PM-induced damage [50]. This implies that the addition of antioxidants into cells or organisms is advantageous, as they reduce the impact of PM on health. Fullerene is a stronger antioxidant with anti-aging properties. Recently, Baati et al. [51] showed that the repeated oral administration of fullerene (in olive oil) in rats doubled their lifespan without causing chronic toxicity. However, a lower water solubility limited the medical applications of fullerene. Hydroxylated fullerenes (fullerenols) represent a major group of fullerene (C_60_) derivatives [52]. Although the hydroxylated form of fullerenes (fullerenols) significantly differs from fullerenes (C_60_) with regard to water solubility, the adaptogenic and biophysical effects, including their interaction with mitochondria, are similar [52]. Moreover, the changes in the solubility and velocity of fullerenols might affect their intra-membrane translocation; hence, their bio-activities/bio-functions are difficult to predict, specifically in complicated culture media or body fluids.

The Standard Reference Material^®^ 1649b (SRM 1649b) has been certified by the National Institute of Standards and Technology for organic constituents (such as polycyclic aromatic hydrocarbons and polychlorinated biphenyl) and mimics the effects of damage attributable to urban dust on skin cells in the current study. We used 1 μM of fullerenol C_60_(OH)_36_ and non-water soluble C_60_(H_2_O) to investigate and compare the levels of inflammation and oxidative stress induced by exposure to PM (1949b). We first demonstrated that fullerenol C_60_(OH)_36_ is partially responsible for the anti-PM-induced early apoptosis and cytotoxicity-linked generation of ROS and the activation of mitogen-activated protein kinase (MAPK) and Akt pathways in HaCaT cells. The HaCaT cell was first generated by Boukamp et al., [53] and is a useful and stable cell platform for disease research and medicine development [10,23,24,54]. The MAPK is a superfamily that includes ERKs, JNKs, and p38 kinases, the three main protein kinase families. Each of these has been shown to play important roles in various processes such as intracellular metabolism regulation, gene expression and integral activities, such as mitosis, differentiation, apoptosis, gene expression, and cellular responses to external stresses and disease [55]. Our previous studies have suggested that the PM-stimulated phosphorylation of ERK1/2, JNK1/2 and p38 was mediated by ROS in HaCaT cells, while the phosphorylation of ERK1/2 simultaneously played a necessary role in downstream p38 phosphorylation [10,24]. In general, the levels of ERK2 were higher than those of ERK1 due to the strong strength of their proximal promoter in most mammalian tissues [56]. Our findings also support this phenomenon. However, some studies have suggested that ERK1 and 2 may present different regulated patterns after stimulation and play different roles in some cells, under certain experimental conditions [56]; this phenomenon was not observed in our study. In general, JNK1 might act as a positive regulator, while JNK2 exhibits a negative or down-regulatory function under normal conditions in several cell and animal models, and during the proliferation of keratinocytes in mouse skin [57]. However, a previous study suggests that JNK2 might be more important than the JNK1 in skin diseases such as human squamous cell carcinoma [58]. Furthermore, JNK2^−/−^ mice showed a greater resistance to chemically-induced skin cancer than JNK1^−/−^ mice [59], which might imply that JNK2 is more sensitive to some chemical agents-induced skin damage, but the details need further investigation. In addition, the functions of Akt are associated with skin aging [60] and are also known to play a prominent role in the healing of wounds and tissue regeneration [61,62]. The thinning of the epidermal layer and delayed development of hair follicles were observed in Akt1-deficient mice [63]. The evidence described above indicates that both MAPK and Akt pathways are essential for maintaining the normal functions of skin cells and facilitate the resistance towards the impact of environmental toxins. Hence, the maintenance of MAPK and Akt expression within the physiological range is advantageous for maintaining skin health. In this study, the PM-triggered higher expression of MAPK and Akt activities could be brought to the normal range (as compared to control group) using pre-treated fullerenol C_60_(OH)_36_, but this could not be achieved with C_60_(H_2_O) (Figure 6); this finding supports our hypothesis.

The expression of various inflammatory proteins has been linked with ROS and downstream MAPK pathways in keratinocytes. Our previous study showed that PM-induced ROS triggered MAPK signal activation, which mediated the expression of COX-2/PGE2 and MMP-9 inflammatory proteins [24]. In addition, the levels of HO-1, ICAM-1, and cPLA2 were also increased because of ROS during inflammation in various types of cells [64,65,66,67]. The up-regulation of these inflammatory proteins might enable the cell to act against the impairments attributable to ROS generation. For example, oxidative or nitrosative stress-induced higher levels of HO-1 were observed to perform antioxidant and anti-inflammatory roles in cells [68,69,70]. In addition, ICAM-1 was highly expressed in epidermal keratinocytes exhibiting lymphoid infiltration and plays an important inflammatory role in transendothelial lymphocyte migration [71,72,73]. Moreover, cPLA2, an enzyme with an inflammatory role, could hydrolyze phospholipids, to produce arachidonic acid or lysophospholipids, which increase COX-2 expression [74,75]; the inhibition of cPLA2 could suppress inflammation in skin diseases [76,77]. The results of the current study revealed that the PM-triggered up-regulation of COX-2, HO-1, MMP-9, ICAM-1, cPLA2 expression and PGE2 production could be inhibited by pretreatment with fullerenol C_60_(OH)_36_, but not with C_60_(H_2_O) (Figure 7). The filaggrin, repetin, involucrin, and loricrin present in keratinocytes have barrier/protective functions, such as the defense against external environmental pathogens and allergen entry or chemical damage, and decrease the level of water loss occurring from the skin [78]. The maintenance of filaggrin, repetin, involucrin, and loricrin levels allows keratinocytes to resist PM-induced cell toxicity [10,33]. The expression of filaggrin is associated with the activation of the inflammatory pathway [79]. Our previous data showed that the increase in COX-2/PGE2 signals negatively regulated filaggrin expression after PM exposure [10]. Here, we further demonstrate the fact that fullerenol C_60_(OH)_36_ can preserve the levels of these of barrier proteins in keratinocytes after PM exposure (Figure 8).

**Figure 9:**
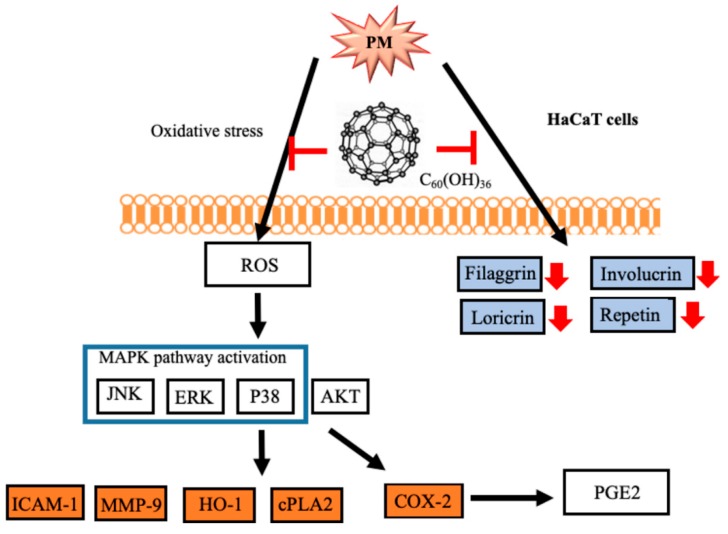
A diagram of the molecular mechanisms underlying the inhibition of PM-induced effects by fullerenols C_60_(OH)_36_ in HaCaT cells. PM causes oxidation, inflammation, and barrier protein losses, which lead to cell toxicity. The levels of ROS were increased and triggered the activation of downstream MAPK and Akt pathways, and subsequently enhanced inflammatory protein (ICAM-1, COX-2, HO-1 and PGE2 etc.) expression. In addition, the loss of barrier proteins after PM exposure could be blocked by fullerenol C_60_(OH)_36_ pre-treatment. This indicates that fullerenols C_60_(OH)_36_ could act via ROS scavenger and anti-inflammatory mechanisms and the maintenance of expression of barrier proteins, to prevent PM-induced adverse effects in HaCaT cells.

In conclusion, this is the first study in which fullerenol C_60_(OH)_36_ reportedly exhibited protective effects against PM-induced oxidative stress and inflammation, and the impairment of barrier proteins and apoptosis, in HaCaT keratinocytes (Figure 9). In contrast, fullerene C_60_ did not exhibit similar effects after PM exposure; this might be attributable to their lower water-soluble properties. Fullerenol C_60_(OH)_36_ could be valuable when used in cosmeceutical products and medicines that inhibit and prevent PM-induced skin injuries in the future.

## 4. Materials and Methods 

### 4.1. Materials

PM (Standard Reference Material^®^ 1649b, SRM-1649b) was obtained from the National Institute of Standards and Technology, Gaithersburg, MD, USA. HaCaT cells were purchased from AddexBio (San Diego, CA, USA). Fullerene, toluene (Tol), tetrabutyl-ammonium hydroxide (TBAH), sodium hydroxide (NaOH), and Sephadex LH-20 resin were purchased from Sigma-Aldrich. All chemicals used in bioassays were of the ACS reagent grade and were purchased from Sigma-Aldrich (St Louis, MO, USA). Buffers and solutions were prepared using Millipore water. The bicinchoninic acid (BCA) protein assay kit was purchased from Pierce (Rockford, IL, USA). Dulbecco’s modified Eagle (DMEM) medium was purchased from GIBCO, Grand Island, NY, USA. Fetal bovine serum (FBS) was obtained from Hazelton Products (Denver, PA, USA). Antibodies were purchased from different sources; total and phosphorylated p38, ERK 1/2, JNK1/2, and phospho-Akt were purchased from Cell Signaling Technology (Danvers, MA, USA); total-Akt and cPLA2 were purchased from Santa Cruz Biotechnology (Dallas, TX, USA); MMP-9, involucrin, and repetin were purchased from Proteintech Group Inc (Rosemont, IL, USA); HO-1 and COX-2 were obtained from Abcam (Cambridge, UK). We also purchased loricrin (Boster, Pleasanton, CA, USA), filaggrin (Genetex, Hsinchu, Taiwan), and GAPDH (Biogenesis, Boumemouth, UK), as well as an immunoblotting enhanced chemiluminescence (ECL) detection kit and Hyperfilms (purchased from GE Healthcare Biosciences, Buckinghamshire, UK).

### 4.2. Thin-Layer Chromatography, Mass and Spectrometer Assay

Analytical thin-layer chromatography (TLC) was performed on precoated plates (Silica Gel 60, Merck). ESI ionization time-of-flight mass (ESI-TOF MS) spectral data were collected on a JMS-T100LP 4G(JEOL) using a mass spectrometer equipped with the ESI source, for detecting positive and negative ions. Typical measurement conditions used were as follows: needle voltage: 2000 kV, orifice 1 voltage: 300 V, ring lens voltage: 10 V, spray temperature: 250 °C. Fourier-transform infrared spectroscopy (FTIR) spectra were recorded using a Thermo Nicolet iS5 FTIR spectrometer.

### 4.3. Transmission Electron Microscopy Assay

Transmission electron microscopy (TEM) was performed using a JEOL microscope (Model JEM-2100) operated at 200 keV, to characterize the sizes and dispersion of the glycofullerenes. A drop of fullerenol solution (~1 μL) was placed on a carbon-coated 200-mesh copper grid. The grid was allowed to dry at room temperature for several hours and further dried under vacuum conditions overnight, prior to TEM analysis.

### 4.4. Hydro-Diameter Size and Zeta Potential

The hydro-diameter size and zeta potential of fullerenol particles were analyzed using a particle analyzer, using the dynamic light scattering (DLS) technique (Horiba, SZ-100V2, Japan) at 25 °C, at a laser angle of 90°. The samples were diluted with pure water. Each value was measured in triplicate.

### 4.5. Synthesis of Fullerenol C_60_(OH)_36_

Fullerene (80 mg, 0.11 mmol) was dissolved in toluene (60 mL), and aqueous NaOH (1 g/mL, 2 mL) and tetrabutylammonium hydroxide solution (TBAH, 1 mL, 40% in H_2_O) were added to this reaction mixture. The reaction mixture was stirred at room temperature in an open flask for 2 h. After the removal of toluene by decantation and concentration under reduced pressure, the brown sludge and remaining solution was stirred with additional water (10 mL), for another 18 h. An additional amount of water (20 mL) was added; the solution was filtered and concentrated. Then, water (5 mL) and MeOH (50 mL) were added to produce a brown precipitate. The precipitate was centrifuged and washed with MeOH (100 mL) and dried in vacuo to obtain fullerenol 2 (48.8 mg, 33%). IR (neat): 3447 (O-H); HRMS (ESI): calculated for C_60_H_36_O_36_ [M + H]^2+^: 666.5533; found: 666.5564.

### 4.6. Cell Culture Conditions

Human epidermal keratinocyte (HaCaT) cells were purchased from AddexBio (San Diego, CA, USA). Cells were cultured in the Dulbecco’s Modified Eagle Medium (DMEM, Gibico, Grand Island, NY, USA) supplemented with 10% FBS (Hazelton Research Products, Denver, PA, USA) and 1% penicillin–streptomycin (Gibico, USA), and incubated at 37 °C in a humidified atmosphere containing 5% CO_2_/95% air. When cultures reached confluence, cells were treated with 0.05% (*w*/*v*) trypsin/0.53 mM EDTA for 5 min at 37 °C. The HaCaT keratinocyte cell line was seeded in a 12-well plate (2 × 10^5^ per well) with 1 mL medium. The cell viability was also simultaneously tested using the 3-(4,5-Dimethylthiazol-2-yl)-2,5-diphenyltetrazolium bromide (MTT) assay, to ensure a survival rate of at least 90% with each passage. After cells were seeded for 24 h for stable, the medium was renewed before the followed experiments were performed. The cells obtained within passages 5 to 12 of HaCaT cells were used for preforming experiments.

### 4.7. Cell Apoptosis Assay

The Annexin V-FITC/propidium iodide assay kit (Thermo Fisher Scientific, Waltham, MA, USA) and caspase-3 assay kit (Santa Cruz Biotechnology, Dallas, TX, USA) were utilized to analyze cell apoptosis by flow cytometry, according to the instructions provided in the manuals. Fullerene and fullerenol (1 μM) were dissolved in pure water and pre-treated to HaCaT cells for 1 h; after treatment with PM (50 μg/cm^2^) for another 24 h, cells were stained with Annexin V-FITC/propidium iodide or added caspase-3 antibody, as suggested in the manuals respectively, and then detected by flow cytometry (Accuri C6, BD Biosciences, San Jose, CA, USA). At least three independent experiments were repeated and data were collected.

### 4.8. Cellular and Mitochondrial ROS Measurement 

The cellular ROS levels were measured using the CellROX and 2′, 7′-dichlorodihydrofluorescein diacetate (H2DCFDA) assays (Thermo Fisher Scientific, USA). Briefly, HaCaT cells were seeded onto 12-well plates (2 × 10^5^ per well) and incubated for 24 h, to ensure that they were stable. Fullerene or fullerenol was added into cells for 1 h, and cells were treated with PM (50 μg/cm^2^) for another 2 h. The fluorescence intensity of the cells was measured using flow cytometry (excitation/mission wavelength, 488/530 nm) after H2DCFDA or CellROX staining. In addition, MitoSOX^TM^ (Molecular Probes, Eugene, OR, USA) was used for detecting the ROS level in the mitochondria using the same treatment schedule described above. The fluorescence intensity was measured at the excitation and emission wavelengths (488 and 585 nm, respectively) for flow cytometry. Experiments were repeated at least three times and the data were collected.

### 4.9. Immunoblotting

HaCaT cells were seeded onto 12-well plates (2 × 10^5^ per well) and incubated for 24 h for continued experiments. Fullerene or fullerenol (1 μM) was added to cells for 1 h and then exposed to PM (50 μg/cm^2^) for another 6 h (kinases) or 24 h (inflammatory- and protection-related proteins), respectively. Lysis buffer was used to lyse cells for protein extraction. Samples with equal amounts of proteins were separated through SDS-polyacrylamide gel electrophoresis (10%–12.5% polyacrylamide) and then transferred onto a polyvinylidene fluoride membrane (Millipore). The primary antibodies, total and phospho-p38, ERK, JNK, Akt, cPLA2, MMP-9, involucrin, repetin, HO-1, COX-2, loricrin, and filaggrin (the suppliers of antibodies have been indicated in 4.1 Materials) were utilized to measure the protein expression levels. The enhanced chemiluminescence (ECL) detection kit was used for measuring signals with a ChemiDocTM XRS+ image system (Bio-Rad Laboratories, Hercules, CA, USA). The blots were then stripped and re-probed using anti-GAPDH for quantitative control analysis. Data were collected from at least three independent experiments exhibiting a similar pattern. The values were calculated from the collected blots and a figure with a closer pattern of statistical values was selected for presentation.

### 4.10. Prostaglandin E2 (PGE2) Production Measurement

HaCaT cells were cultured in a 12-well plate for 24 h before the addition of fullerene or fullerenol (1 μM). After they received treatment for 1 h, cells were exposed to PM for another 24 h. The medium was collected for further measuring PGE2 levels using the PGE2 enzyme immunoassay kit (Cayman Chemical, Ann Arbor, MI, USA), as per the manufacturer’s instructions. Three independent experiments were performed and values were calculated.

### 4.11. Statistical Analysis

All data were expressed as mean ± SEM values and analyzed using GraphPad Prism software (v5, GraphPad, San Diego, CA, USA), with one-way ANOVA, followed by the post-hoc Tukey’s multiple comparison (multiple groups) test. A *p* value < 0.05 was considered significant.

## Abbreviations

COX-2	Cyclooxygenase
cPLA2	Cytosolic phospholipases A2
ERK	Extracellular regulated protein kinase
FITC	Fluorescein isothiocyanate
GAPDH	Glyceraldehyde 3-phosphate dehydrogenase
HO-1	Heme oxygenase-1
ICAM-1	Intercellular adhesion molecular-1
JNK	c-Jun N-terminal kinase
MAPK	Mitogen-activated protein kinase
MMP-9	Metalloproteinase-9
PM	Particulate matter
PGE2	Prostaglandin E2
ROS	Reactive oxygen species
